# Contactless Interface Using Exhaled Breath and Thermal Imaging

**DOI:** 10.3390/s23073601

**Published:** 2023-03-30

**Authors:** Kanghoon Lee, Jong-Il Park

**Affiliations:** 1 Department of IT Engineering, Sookmyung Women’s University, Seoul 04310, Republic of Korea; aeternalis999@gmail.com; 2 Department of Computer Science, Hanyang University, Seoul 04763, Republic of Korea

**Keywords:** computer interface, thermal conduction, exhaled breath

## Abstract

A new type of interface using a conduction hot spot reflecting the user’s intention is presented. Conventional methods using fingertips to generate conduction hot points cannot be applied to those who have difficulty using their hands or cold hands. In order to overcome this problem, an exhaling interaction using a hollow rod is proposed and extensively analyzed in this paper. A preliminary study on exhaling interaction demonstrated the possibility of the method. This paper is an attempt to develop and extend the concept and provide the necessary information for properly implementing the interaction method. We have repeatedly performed conduction hot-point-generation experiments on various materials that can replace walls or screens to make wide use of the proposed interfaces. Furthermore, a lot of experiments have been conducted in different seasons, considering that the surface temperature of objects also changes depending on the season. Based on the results of an extensive amount of experiments, we provide key observations on important factors such as material, season, and user condition, which should be considered for realizing contactless exhaling interfaces.

## 1. Introduction

An interface system is necessary for a person to operate any mechanical device. This type of system can be divided into two categories: manipulating with fingers or recognizing hand motions. Studies utilizing cameras and touchscreen devices have been carried out regarding the technique of manipulating objects with one’s fingers. This particular paper focuses on a camera-based interface method. Studies utilizing cameras have demonstrated that touch interfaces based on devices equipped with RGB (visible light), near-infrared, and depth cameras can lead to user actions that are either unintended or not possible in particular usage environments.

There is a touch interface that uses a far-infrared ray (thermal imaging camera) camera as a method for being robust to a user environment and accurately recognizing a user’s finger contact [1,2,3,4,5,6]. This approach involves touching an object’s surface with a finger, which causes body heat from the finger to transfer to the surface and create a hot spot. A thermal imaging camera can be used to detect residual hot spots on an object’s surface, which can then be recognized as a touch point, making it a form of interface. As these points are produced by the user’s direct finger action on an object’s surface, a touch response will not happen without contact. Moreover, indoor lighting changes do not influence far-infrared rays. These features have led to the use of thermal imaging cameras in research, along with motion sensors and infrared rays [7,8,9,10].

The interface using the thermal imaging camera has the advantage that other camera sensor methods are robust against environmental characteristics or detection errors. However, due to its high price and lack of widespread use, there have been relatively few follow-up or related studies. Therefore, it is essential to conduct ongoing research, not only on novel techniques, but also on follow-up or related studies. Previous research was carried out assuming that a user’s finger would generate a hot spot on the material’s surface, and only one type of material was used in the investigation [1,2,3,4,5,6]. This means that the material used in that study was effective in producing hot spots when subjected to a user’s finger. Nonetheless, it is important to note that not all users can generate hot spots with equal efficiencies and that the ability to create hot spots varies considerably depending on the material used.

We performed a study using exhalation instead of hands to improve the hot spot interface created by fingers [11,12]. This paper is a continuation of that study, which aimed to organize the benefits and features of interfaces that utilize exhaled breath and perform different experiments to support them. The experiment involved analyzing the temperature information of the conduction hot spot and its location obtained by a thermal imaging camera, based on material and season. The experiment revealed issues with the current method, which was unsuitable for users based on their physical condition. It also demonstrated the effectiveness of a new approach that utilizes exhaled breath. Additionally, the study analyzed data on the creation of hot spots in various materials that were previously unavailable or insufficiently examined in thermal imaging studies with a focus on potential applications.

This thesis will present fresh methods and information pertaining to interface research to be utilized by researchers in related fields. It will offer a chance to apply and employ these methods. Specifically, analysis of the hot spots generated by fingertip contact and exhalation on different materials can be referenced and integrated into various studies that employ thermal imaging cameras.

The remainder of this paper is organized as follows. Section 2 describes related studies utilizing camera-based touch interfaces and thermal imaging cameras. Section 3 discusses the method of using exhalation breath as an interface and ways to address its drawbacks. In Section 4, experiments are carried out to create residual hot spots in different materials with the aim of improving usability. Lastly, Section 5 provides a summary of the findings and outlines future research directions.

## 2. Related Work

Numerous studies have been conducted regarding the utilization of hands as a method of interaction in interfaces. These interfaces can be broadly categorized as hand gesture recognition interfaces and touch interfaces that are operated using fingers. Methods for recognizing hand movements include using a color glove [13], using color bands on fingers [14], and inferring hand and finger movements by wearing an IMU (inertial measurement unit) on the wrist and fusing the results with EMG (electromyography) signals from forearm muscles [15]. Using these methods requires the drawback of wearing a distinct device. Furthermore, these methods involve individual devices that are meant for personal use only. Consequently, devices that can be utilized for multiple persons, rather than for personal use, are incredibly advantageous.

As a method using a camera, the hand shape is analyzed and tracked using the background and the skin color of the other hand [16]. The method of using depth sensors increases the accuracy of the hand pose by separating the background and the hand according to the distance [17]. Various studies have been conducted, such as the method of combining RGB and ToF (time-of-flight) cameras [18]. However, the method that employs both an RGB and depth camera with infrared light is prone to lighting changes and has depth measurement errors due to sensor noise.

A touchscreen interface is commonly found on small mobile devices and monitors, where a finger can be used for input. However, the installation of large touchscreens is technically challenging and expensive, and their weight and volume can limit their use in certain locations. Additionally, some alternative methods using projectors and electrical signals can be used instead of touchscreens; however, these are susceptible to lighting changes and require complex hardware configurations, making them less practical for widespread use [19,20]. The projector–camera system is a popular method of studying camera-based touch interfaces. While RGB cameras are commonly used due to their accessibility, they face challenges in detecting hand outlines or distinguishing skin color in low or high light conditions. This results in unstable finger contact recognition [21].

An RGB camera can capture visible light, while an infrared camera is designed to capture the infrared region of the electromagnetic spectrum. Infrared cameras are robust against changes in lighting and can be used in dark environments because they capture infrared rays reflected by objects. To utilize these characteristics, interface studies using projectors or LED displays have also been conducted [22,23]. However, to use an infrared camera, infrared lighting is necessary, and it can be affected by indoor lighting because infrared wavelengths are emitted even in such lighting. Additionally, determining finger touch can be inaccurate.

Thermal imaging cameras have been extensively utilized for military applications, but more recently they have also found their way into the industrial sector [24]. These cameras operate by detecting far-infrared wavelengths emitted by objects, measuring the amount of thermal energy present in those wavelengths, and then displaying those data as a visual image. The advantage of these cameras is their ability to visualize heat energy, which is invisible to the naked eye, in both bright and dark settings. These features are used in various fields such as forest fire detection [25], fire detection monitoring [26], soil moisture estimation [27], solar panel detection [28], medical multi-vital sign measurement [29], and respiratory analysis [30].

Numerous research studies have been conducted on the use of thermal imaging cameras, which offer several benefits, in motion recognition and touch interface research. This is due to the fact that the shape of a person is distinctly displayed in the thermal image by the temperature variation with the surrounding objects. Various approaches have been explored, such as motion recognition interaction using the entire human body [7], dividing the human body for multi-modal usage [8], and motion recognition techniques that utilize the far-infrared rays reflected by materials [9,10], among others.

For finger touch, which is not a motion or gesture recognition, research has been conducted to utilize the hot spot created by contacting a finger with the surface of an object by using the heat conduction phenomenon caused by the temperature difference between the two objects. A typical example is a study that recognizes a conductive hot spot via finger contact by combining a projection camera system and a thermal imaging camera. Some such studies include a study using a table as a surface [1]; “TurboTablet” [2] which projects a project onto a transparent screen; “HeatWave” [3], which further developed the interaction for recognizing the conduction hot spot on the surface of an object; and a study using a bathroom curtain [6]. Examples of studies that combined a thermal imaging camera and other devices include “Dante vision” [4], which combined thermal imaging with infrared and depth sensors built into Kinect, and “Thermal Touch” [5], which combined mobile devices.

As such, various studies have been introduced to recognize conduction hot spots generated by finger contact using thermal imaging cameras. However, the focus has been on the feasibility of this method, and there is a lack of research on the conduction hot spot of objects. Moreover, some individuals may be unable to use the finger contact approach. This paper presents an interface based on a conduction hot spot that is accessible to a broader population. We conduct multiple experiments to examine the conduction hot spot generated on object surfaces and present our findings.

## 3. Interface Using Thermal Image

### 3.1. Fingertip Touch Interface

Thermal imaging interfaces utilize the detection of residual heat or conduction hot spots on the surface of objects, which are left behind by the fingertips. This is possible because the surface temperature of the interface object is lower than that of the human body, and, therefore, various types of objects can be used for this purpose. Using a thermal imaging camera to detect hot spots has several benefits. It can detect such hot spots even in challenging backgrounds or without being influenced by illumination, making it more reliable than visible light or near-infrared camera methods. The precision of thermal imaging cameras reduces as the distance of measurement increases. However, when employed as an interface, the measurement distance typically ranges from 1.5 m to 4 m, sufficient to identify hot spots accurately.

Overall, it is relatively easy to generate and identify a conductive hot spot on an object’s surface by using one’s finger. However, particular individuals may encounter difficulty in producing detectable hot spots. This is due to the fact that individuals with cold hands and feet have lower fingertip temperatures, resulting in smaller and cooler conduction hot spots. While it may be possible to adjust the threshold value range used in the detection algorithm to identify these spots, this may lead to an increased risk of false detection.

**Figure 1:**
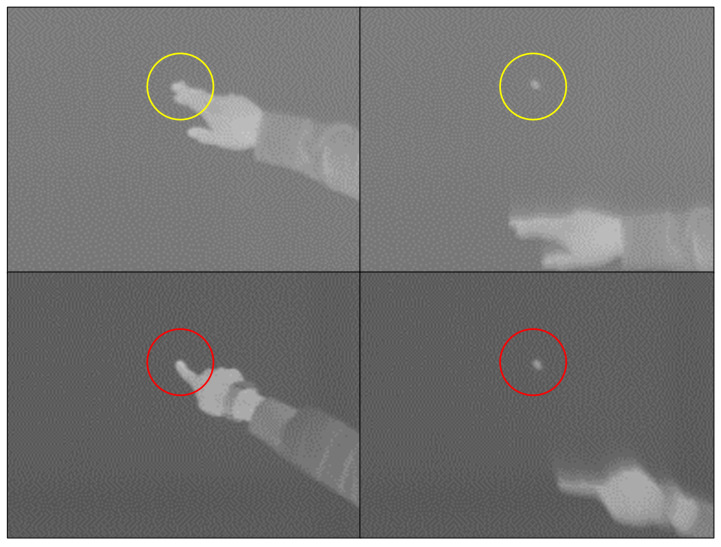
Comparison of fingertip touch conduction hot spot. (Material: canvas paper for oil painting) **Top**: upper part of the finger 33.27 degrees, hot point 29.94 degrees, surface 24.72 degrees. (Yellow circle: touch area) **Bottom**: upper part of the finger 32.06 degrees, hot point 27.81 degrees, surface 21.23 degrees. (Red circle: touch area).

To compare the variation in the temperature of the conduction hot spot based on individuals, the fingertip was used to create the hot spot on the canvas paper under identical environmental conditions. Figure 1 is a thermal image of a conduction hot spot created by two people on the same material during winter. There is a difference of more than 2 degrees between the conduction hot spot temperatures in Figure 1 (top) and (bottom) and a difference of more than 3.5 degrees in the surface temperature.

Participants with low hot spot temperatures reported that their hands or feet were cold. The variance in the conduction hot spot’s temperature is impacted by the duration and pressure of the participant’s finger on the surface; it is primarily affected by the temperature of the individual’s finger. For this reason, a new approach is needed to generate the conduction hot spot due to the possibility of encountering issues in both creating and identifying it using a finger.

### 3.2. Method by Exhalation

During winter, individuals with cold hands and feet tend to experience significantly lower temperatures in their exposed fingers. Nonetheless, the human body maintains a constant overall body temperature, regardless of whether an individual has cold hands or not. Typically, a person’s breath is as warm as their body temperature, which is generally higher than the temperature of their fingers. From this, it can be inferred that when a conduction hot spot is created on the surface of an object by exhalation, the temperature is generally higher than the surface temperature. Therefore, when a conduction hot spot is generated on the surface of an object due to respiration, the location of the conduction hot spot can be detected by the surface temperature difference. However, the shape of the conduction hot spot due to expiratory breathing is large and widely spread, and the temperature is lower than expected. The reason for this is the difference in the exhalation method.

**Figure 2:**
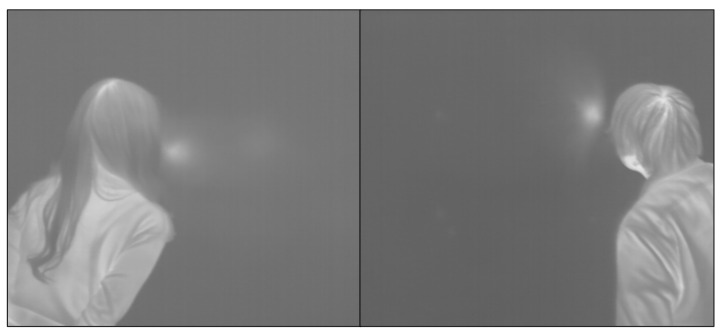
Form of conduction hot spot by exhalation breath.

People’s exhaled breath can be categorized into two types based on the exhalation speed: slow exhalation generates warm breath, while fast exhalation produces windy breath. The temperature and size of the hot spot generated on the object’s surface differ depending on the exhalation method. This corresponds to the distinction between exhaling slowly and quickly. Figure 2 displays the conduction hot spots produced by exhalation using both methods. Since people breathe out unconsciously, the temperature deviation of the conduction hot spot due to the breath is large, and the exhaled breath spreads wider, similar to a cone, as the object’s surface is farther away and tilted.

In the case of exhalation, the temperature difference according to the person can be reduced, but errors regarding the temperature difference and the generation position of the conduction hot spot occur due to the various factors described above. The job of the creation position involves operating the interface and is comparable to the touch point detection area on a touchscreen. As a result, there is a requirement for a technique that can produce a hot spot of appropriate size at a location designated by the user.

### 3.3. Exhalation Breath Interface Using a Hollow Rod

In a previous study, a hollow rod was used to gather the breath in one place without spreading [11,12]. Utilizing a hollow rod presents numerous benefits. Firstly, when breathing onto a surface using a hollow rod, the surface temperature at the conduction hot spot increases as the heat energy from the mouth is focused instead of diffusing into the air. Additionally, the circular hot spot produced is smaller and more concentrated, resulting in improved detection performance.

**Figure 3:**
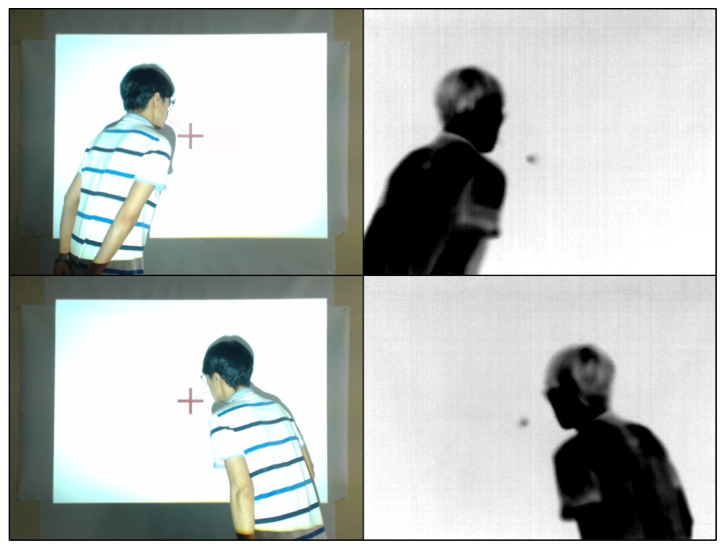
A scene of exhalation using a hollow rod and the resulting thermal image of a conductive hot spot. If you exhale over the cross mark, it will show up as a black dot on the thermal image. (**Left**: RGB image, **right**: thermal image).

Second, a conduction hot spot can be created at a specific location by generating it at a distance equal to the length of the hollow rod. This is a more precise method compared to just exhaling. An experiment was carried out to confirm this, where a projector was used to project an image with a “cross” mark and a conduction hot spot was generated precisely on the mark. In Figure 3, a projector projects a target grid to create a conductive hot spot at a specific location. In the experiment, participants were tasked with generating a conduction hot spot at the “cross” mark using three different methods. The use of a hollow rod produced a hot spot that was closer to the marked position compared to the simple exhalation method.

Third, people with cold hands struggled to produce hot spot areas when touching with their fingertips. However, even for people with cold hands, the temperature of the breath exhaled from their mouths is almost the same as that of others, such as body temperature. Therefore, using hollow bars can facilitate the creation of high-temperature hot spots. This implies that individuals with cold hands can operate the interface without difficulty by utilizing the conduction hot spot.

**Figure 4:**
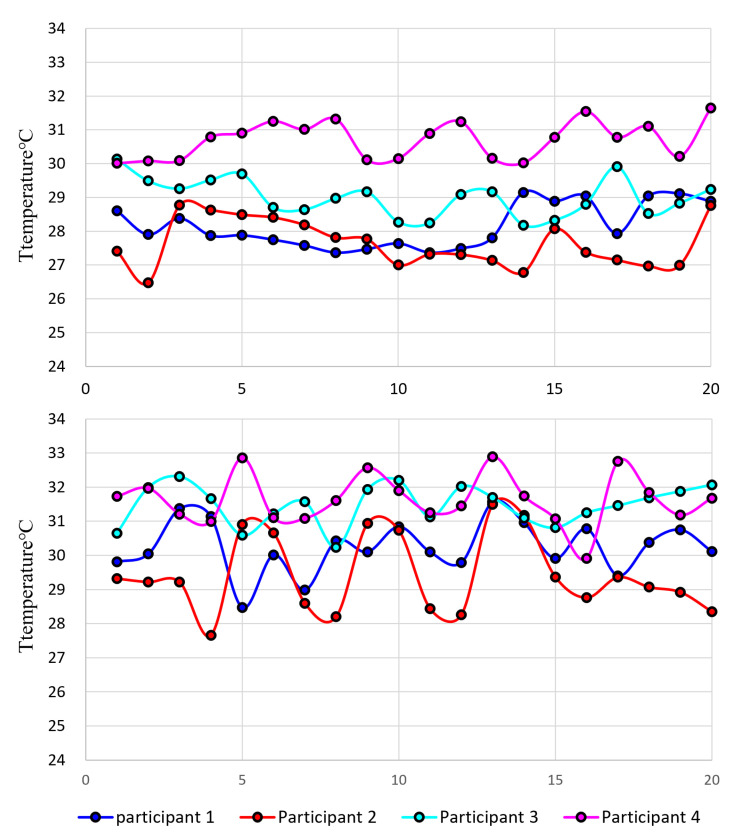
Graph of hot spot temperature by participant (Creation method: **Top**: exhalation using a hollow rod, **bottom**: fingertip touch) (Participant2: cold constitution of hands and feet).

Figure 4 illustrates a comparison of the temperatures of the conduction hot spots generated on a material’s surface using different methods. The top section of Figure 4 shows the temperature generated by contacting the fingertips, while the bottom section shows the temperature generated by exhaling the breath through a hollow rod.

The significant temperature difference observed in the hollow rod method is attributed to variations in exhalation time and intensity. To ensure ease of use, participants were given a brief explanation of how to exhale and instructed to exhale for 2 to 3 s. This experiment should be conducted in a comfortable manner since strict control may cause inconvenience to users and inaccurately reflect real-life usage. Examining the graph, it is evident that utilizing the hollow rod resulted in an increase in the maximum and minimum temperatures of the hot spot. Moreover, the hot spot temperature for both groups was higher than that of the hot spot generated using fingertips. Consequently, thermal imaging revealed the hot spots more prominently.

This method of detecting hot spots can serve as a substitute for touch points in camera-based touch interfaces and can be used in conjunction with projector–camera systems. By utilizing a hollow stick to create hot spots, a person with cold hands or limited hand mobility can use this technology. Moreover, since touch points are represented by hot spots, persons who can use their fingers can also interact with the system, enabling various users to utilize the same interface. Notably, this system is adaptable for use in both table-top structures and large-scale exhibition spaces and is highly portable and easy to install. By employing materials that facilitate the creation of hot spots, this interface has the potential to replace bulky touch screens.

## 4. Comparison and Analysis of Conduction Hot Spot Generation for Utilization

### 4.1. Conduction Hot Point Generation Experiment by Material

To utilize the exhale breath interface in various places, hot spot generation information according to the material is required. An experiment was conducted to create hot spots in an indoor office space during winter and summer between 7:00 and 9:00 p.m. The lowest indoor temperature recorded during the experiment was 17.5 degrees Celsius in winter and 27 degrees Celsius in summer. The experiment is not to measure the exact heat conduction value according to the material but to measure the hot spot temperature for each material in a general indoor environment and use it to estimate the threshold value for detection. The thermal imaging camera used in the experiment was a VarioCAM hr head 420 model, and the resolution of the thermal imaging camera was 384 × 288 autofocus pixels. The distance between the thermal imaging camera and the material surface is 2–3 m.

**Figure 5:**
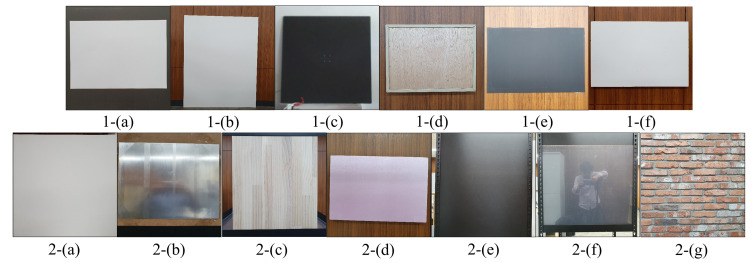
First experiment material: 1-(**a**) coated paper, 1-(**b**) canvas paper for oil painting, 1-(**c**) iron plate, 1-(**d**) MDF in which small wood particles were united, 1-(**e**) acrylic (thickness 2 mm), and 1-(**f**) styrofoam board (thickness 5 mm). Second experiment material: 2-(**a**) canvas paper for oil painting, 2-(**b**) aluminum (thickness 2 mm), 2-(**c**) radiata pine wood, 2-(**d**) styrofoam board used as a hot insulating material (thickness 30 mm), 2-(**e**) pomex: PVC foam sheet (thickness 2 mm), 2-(**f**) transparent acrylic (thickness 5 mm), and 2-(**g**) red brick.

The thermal video was shown to the experiment participants, and the method of generating the conduction hot spot was explained. The fingertip contact time and exhalation time were set to last 2 s. During the experiment, the duration did not exceed the maximum of 3 s and was at least 1.5 s. A total of 11 materials were utilized in two experiments; 6 materials were used in the first experiment, which took place during the winter month of December, and 7 materials were used in the second experiment, which was conducted indoors during the summer months of June and July (see Figure 5).

The temperature of the hot spot area was measured as soon as the fingertip moved away from the object’s surface and the hot spot became visible. Similarly, when exhalation stopped, the temperature was measured from the point at which the hot spots were observable.

The conductive hot spot was created by dividing the object’s surface into quadrants and performing the process of forming the hot spot 30 to 35 times with intervals of around 30 to 40 s. Instead of creating the hot spot repeatedly at the same location, the surface was divided into quadrants to create them in different locations. This was carried out because the participants created the hot spots through various movements, such as body movement, finger contact, or exhaling, and they were not repeated in the same way.

**Figure 6:**
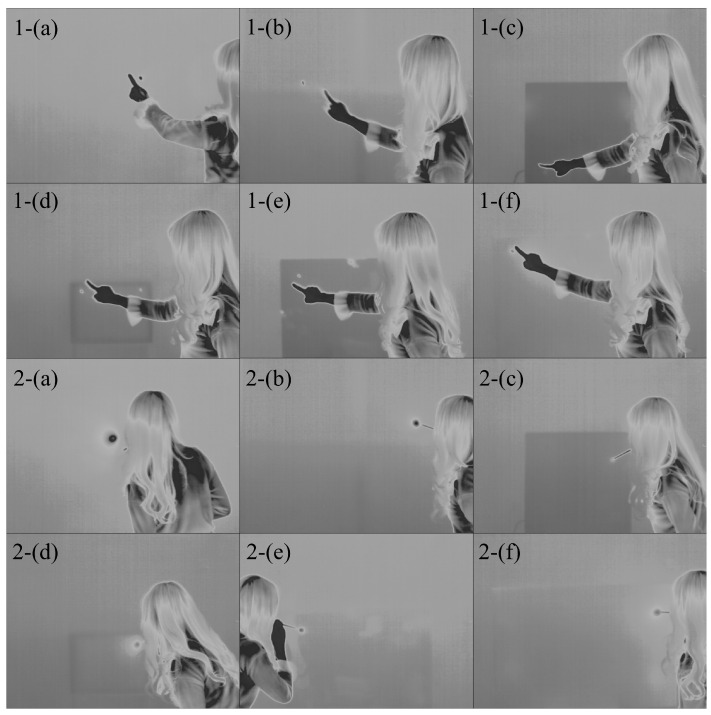
Scene of creation of conductive hot spot by material (thermal image). Method: 1-fingertip contact, 2-exhalation using a hollow rod. Material: (**a**) coated paper, (**b**) canvas paper for oil painting, (**c**) iron plate, (**d**) MDF in which small wood particles were united, (**e**) acrylic (thickness 2 mm), and (**f**) styrofoam board (thickness 5 mm).

Figure 6 displays thermal imaging photographs that illustrate two methods for generating conduction hot spots for each material. The experimenters attempted to control the hollow rod with their mouths alone, without using their hands whenever possible. The images in Figure 6 depict hot spots generated without holding the hollow rod. Exhaling directly onto the surface caused issues with accuracy due to visual errors. However, using a 25 cm hollow rod allowed the experimenters to generate a hot spot at the intended location without any visual errors.

**Figure 7:**
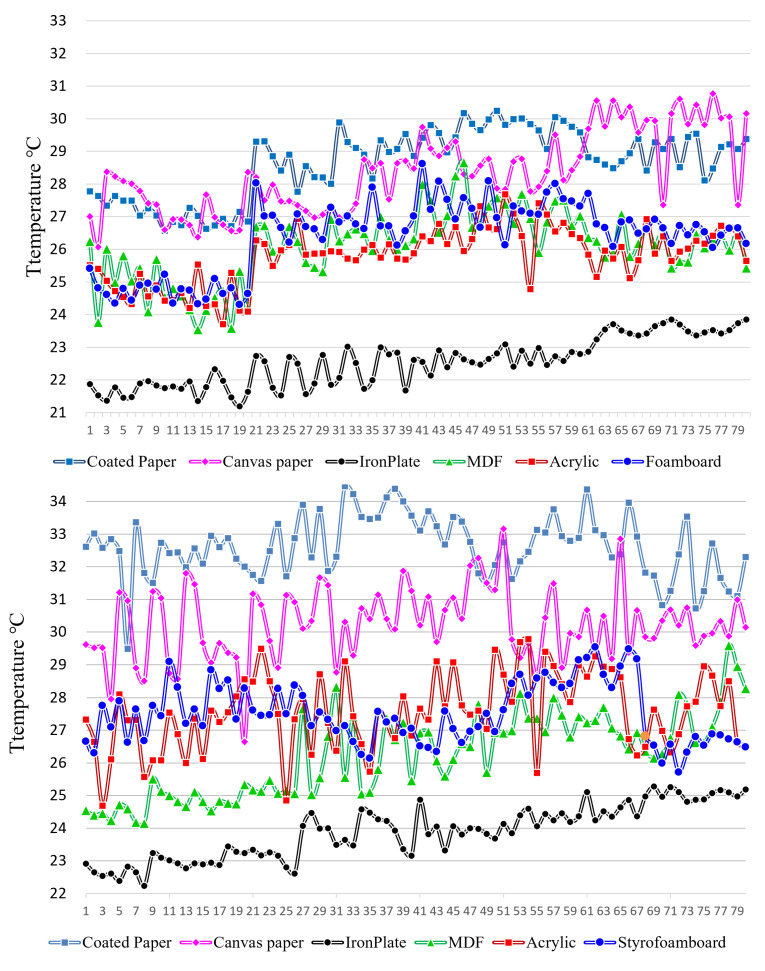
First experiment hot spot temperature graph for each material. Creation method: **top**: fingertip touch, **bottom**: exhalation using a hollow rod (vertical axis: celsius temperature, horizontal axis: temperature data of participants).

Coated and canvas paper exhibit elevated temperatures compared to other types of materials, irrespective of the method used to generate hot spots. In the case of the iron plate, the heat of the breath was visible when exhaling, but the hot spot was not properly created on the surface. The maximum temperature at the center of the conduction hot spots generated through such repeated experiments was measured and shown as a graph of the surface conduction hot spot temperature of the materials, as shown in Figure 7. During the initial trial, each participant produced 80 hot spots by repeating the process 20 times for each position located in the quadrant of the object. The temperature readings were taken and averaged every four times and then grouped into 20 items per participant. The graph’s horizontal axis displays the temperature data for 80 hot spots from four participants in sequence.

Coated and canvas paper exhibit elevated temperatures compared to other types of materials, irrespective of the method used to generate hot spots. In the case of the iron plate, the heat of the breath was visible when exhaling, but the hot spot was not properly created on the surface. Acrylic generally has a lower hot spot temperature than steel plates, but it is a versatile material that can be used in many applications. By using a hollow bar, its performance can be comparable to or even better than that of a foam board or MDF. However, it is important to note that, in the experiment, the acrylic used was only 2 mm thick. On the other hand, the surface temperature of the iron plate remains relatively low in comparison to other objects due to its high thermal conductivity. This means that increasing its temperature by simply touching or breathing on it for a few seconds is difficult.

**Figure 8:**
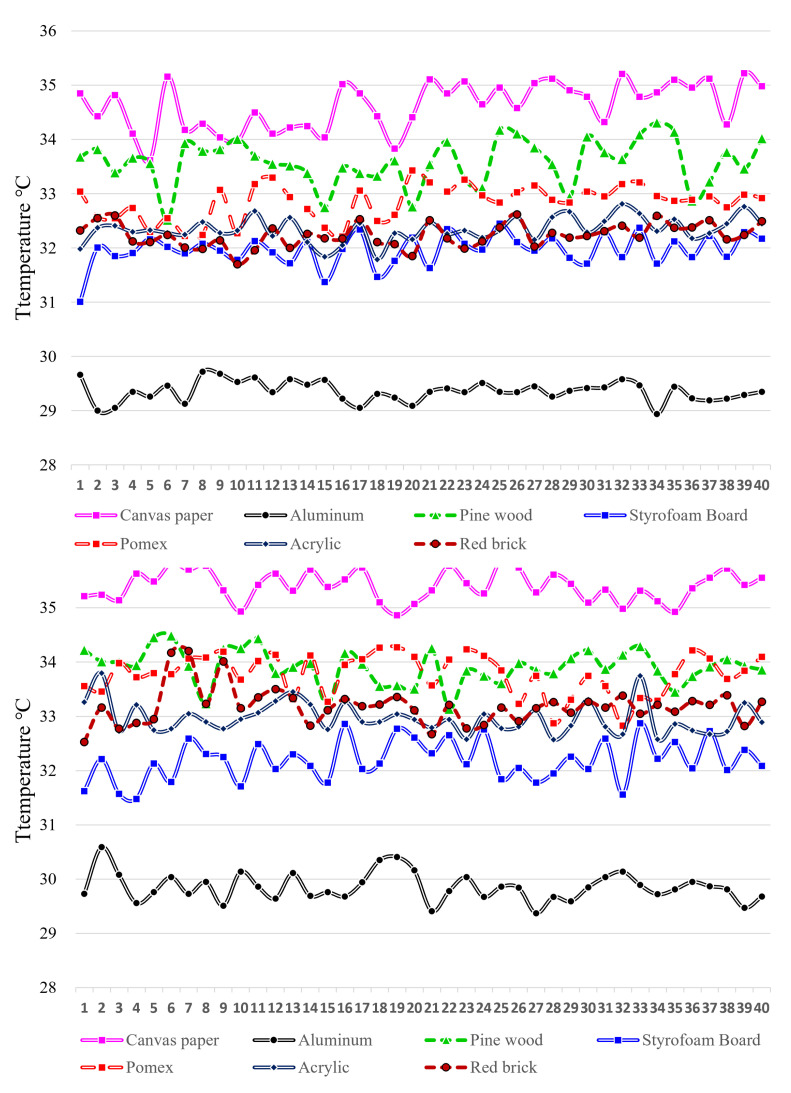
Second experiment hot spot temperature graph for each material. Creation method: **top**: fingertip, touch, **bottom**: exhalation using a hollow rod (vertical axis: celsius temperature, horizontal axis: temperature data of participants).

The reason for conducting the second experiment in June is because indoor temperatures vary depending on the season, and because the surface temperature of materials also changes with the indoor temperature, the temperature of the conduction hot spot will also change. The second experiment was conducted in the same way as the first experiment, and there were two participants. Figure 8 shows the measured hot spot temperature as a graph.

The initial test revealed that aluminum, similar to the steel plate, had a hot spot temperature that was remarkably low, and the difference in temperature with the material surface was under 0.5 degrees Celsius. Conversely, canvas paper and pinewood exhibited a high temperature distribution, as shown in Figure 8-top. When using a hollow rod to generate a conductive hot spot, it was observed that aluminum had a low temperature, and no significant difference was observed from the method of contacting a fingertip. However, in the case of canvas, pine, and foam board, the temperature of the conduction hot spot exceeded 34 degrees Celsius, as depicted in Figure 8-bottom.

### 4.2. Conduction Hot Spot Analysis by Materials

The experiment to measure the temperature of the hot spot during conduction for each material was conducted in a regular office space instead of a highly controlled laboratory setting. Moreover, due to the thickness of the materials being in the order of several mm, it cannot be claimed that the thermal conductivity of the material is accurately represented. Besides thermal conductivity, the temperature of the conduction hot spot is affected by various factors, such as the characteristics of the thermal imaging camera’s sensor, the material’s surface condition, and the temperature and humidity of the surrounding space, making it a complex reflection of several variables [31].

Looking at the graphs in Figure 7 and Figure 8, it can be seen that the conduction hot spot temperature of some materials is low. This is because the thermal conductivity is different depending on the material. Thermal conductivity is the hot transfer characteristic of a material with a thickness of 1 m at a pressure of 1 atmosphere. Generating a conduction hot spot in metals such as iron, stainless steel, and aluminum is challenging due to their high thermal conductivity. In the experiment, the temperature of the hot spot was comparatively lower in aluminum as it has a high thermal conductivity. Conversely, materials such as cotton or paper have low thermal conductivity compared to metals [31,32,33].

**Figure 9:**
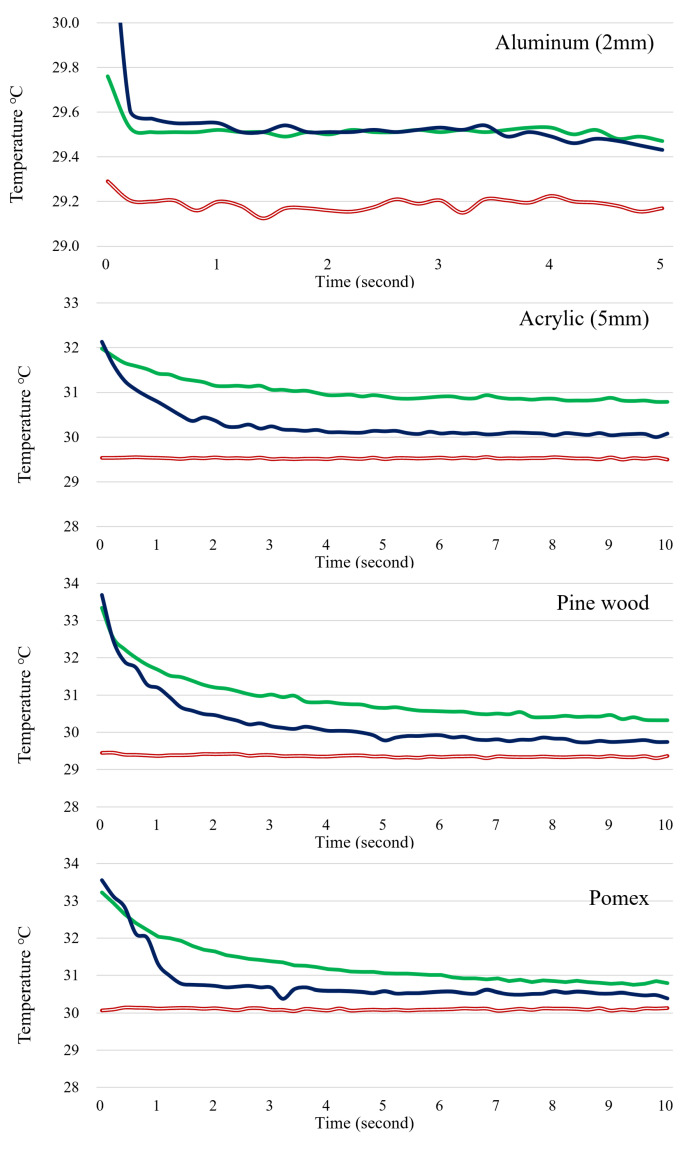
Temperature change over time after hot spot creation. Navy blue line: hollow rod; green line: finger touch; red line: surface of a material.

The graph in Figure 9 illustrates the temperature change over time of the hot spot caused by conduction on the material’s surface. The green line represents the temperature of the contact point with a fingertip, the navy blue line depicts the temperature when blowing air through a hollow bar, and the black line represents the surface temperature of the material. Typically, the measurement lasted from 5 to 10 s, except for aluminum and stainless steel, which made it difficult to differentiate between the material’s surface and the conduction hot spot temperature using thermal imaging due to minimal temperature differences. Hence, the measurement duration was reduced to approximately 3 to 5 s. The high material surface temperature was due to the testing conducted in June, a hot month similar to summer, with the air conditioner turned off in the usage environment test.

Observing the aluminum chart reveals that the temperature gap between the surface temperature and the conduction hot spot ranges from 0.5 to 0.7 degrees. Additionally, there is a rapid temperature drop within half a second. Although it appears that the temperature difference is sustained, it is important to consider that the thermal image may contain a temperature error of around 0.5 degrees due to noise. Consequently, any hot spots cannot be distinguished visually or detected using blob-detection methods.

Acrylic, pinewood, and pomax have the ability to identify areas of high temperature as a result of hot spots that exhibit temperatures of more than 2 degrees Celsius higher than the surrounding surface. Within a time frame of around 0.5 s of the creation of a hot spot, there is a minor variation in the detection sensitivity among these materials, with fingertip contact typically resulting in a higher temperature reading compared to using a hollow rod. This disparity is believed to stem from the fact that direct contact has a greater impact on an object than heat transfer through the air.

**Figure 10:**
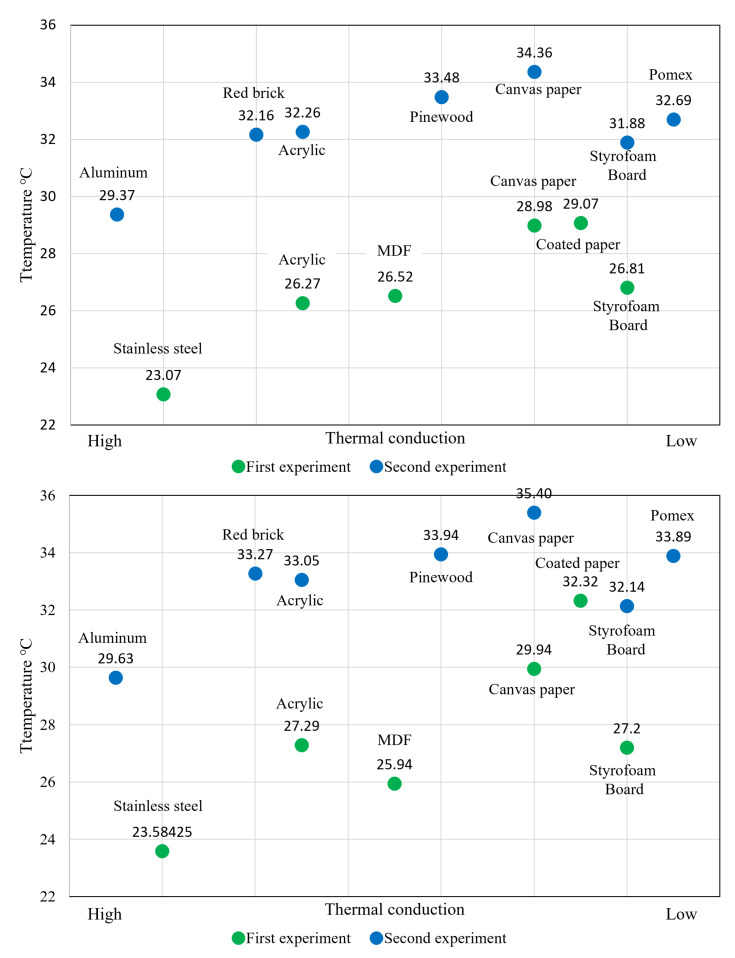
Distribution of conduction hot spot generation temperature according to material group 2 **Top**: fingertip touch, **bottom**: using hollow rod.

The temperatures of the hot spots in the first and second experiments were compared based on the conduction of heat in the materials used. Figure 10-top represents fingertip contact, while Figure 10-bottom represents a hollow bar. A higher average hot spot temperature was observed for materials with lower thermal conductivity. The average temperature of the hot spot was lower during the first experiment conducted in February and higher during the second experiment conducted in June.

### 4.3. Results and Discussion

The experiment testing hot spot generation was conducted in two ways, finding that exhaling through a hollow rod results in a higher hot spot temperature than touching with a finger. Even for individuals whose hands are not cold, the temperature of their hands is still lower than that of exhaled breath due to various factors, such as the temperature difference between body extremities and heat transfer through the skin. Furthermore, when a person touches an object with their hand, the temperature experienced depends not only on the surface temperature of the object but also on its thermal conductivity [33].

Low thermal conductivity materials, such as textile canvas paper and coated paper, may result in a significant rise in hot spot temperature whereas metals with high thermal conductivity were incapable of producing noticeable hot spots [34]. Additionally, during winter, when the object’s surface was cold, high thermal conductivity materials posed a challenge in the experiment because the finger temperature decreased with repeated contact, causing discomfort to the user when touching [35].

In Figure 9, the temperature inversion phenomenon of the hollow rod’s hot spot is not a drawback because the hot spot interfaces are utilized repeatedly rather than only once. Consequently, a brief extinction period following the creation of a hot spot decreases the likelihood of inaccurately detecting the subsequent hot spot’s location.

The experiment revealed that by creating hot spots in different materials, it was possible to verify temperature and seasonal variations based on their thermal conductivity. However, it is not possible for the pressure applied by participants’ fingers, the strength of exhaled breath, and the duration of the hot spot to be identical. Moreover, if the method is applied in practice, users may not be able to produce the same hot spot consistently. To address this issue, adjusting the detection algorithm’s threshold value according to the material used could help reduce errors.

## 5. Conclusions

This paper described the contactless interface technique utilizing thermal imaging cameras and carried out experiments to generate hot spots in different materials for diverse applications. By utilizing breath, hot spots were produced without contact, and hollow bars were used to concentrate the spreading exhaled breath. This approach enabled persons who were previously unable to produce hot spots to generate enough of them and allowed those who could not use their hands to participate. In subsequent research, experiments were conducted to generate hot spots for a range of materials commonly found in daily life based on the potential shown in basic research. Valuable information was obtained through these experiments, confirming the suitability of certain materials for use and identifying seasonal variations. This information can be utilized as a reference for related research.

The contactless interface introduced in this paper has a deviation of the generated hot spots, and there is fatigue and inconvenience when using hollow bars continuously. Furthermore, the experiment on hot spot generation was carried out in various seasons, although the materials used were not entirely consistent except for a few. Future studies plan to find ways to create stable hot spots and improve the ease of use of hollow bars to solve these problems. Additionally, trials will be carried out using identical materials to validate variations in seasonal hot spot shifts. Finally, the interface covered in this paper demonstrated the interface by projecting it on a wall with a projector. There are plans to organize the system in the form of kiosks for broad utilization.

## Data Availability

Not applicable.
